# Topography of Distinct *Staphylococcus aureus* Types in Chronic Wounds of Patients with Epidermolysis Bullosa

**DOI:** 10.1371/journal.pone.0067272

**Published:** 2013-06-25

**Authors:** Magdalena M. van der Kooi-Pol, Mehdi Sadaghian Sadabad, José C. Duipmans, Artur J. Sabat, Tim Stobernack, Till F. Omansen, Gerlinde N. Westerhout-Pluister, Marcel F. Jonkman, Hermie J. M. Harmsen, Jan Maarten van Dijl

**Affiliations:** 1 Department of Medical Microbiology, University of Groningen and University Medical Center Groningen, Groningen, The Netherlands; 2 Department of Dermatology, University of Groningen and University Medical Center Groningen, Groningen, The Netherlands; 3 Laboratory for Infectious Diseases and Perinatal Screening, National Institute for Public Health and the Environment, Bilthoven, The Netherlands; University Hospital Münster, Germany

## Abstract

The opportunistic pathogen *Staphylococcus aureus* is known to interfere with wound healing and represents a significant risk factor for wound infections and invasive disease. It is generally assumed that one individual is predominantly colonized by one *S. aureus* type. Nevertheless, patients with the genetic blistering disease epidermolysis bullosa (EB) often carry multiple *S. aureus* types. We therefore investigated whether different *S. aureus* types are present in individual wounds of EB patients and, if so, how they are spatially distributed. The staphylococcal topography in chronic wounds was mapped by replica-plating of used bandages and subsequent typing of *S. aureus* isolates. Individual chronic wounds of five patients contained up to six different *S. aureus* types. Unexpectedly, distinct *S. aureus* types formed micro-colonies that were located in close proximity and sometimes even overlapped. While some adjacent *S. aureus* isolates were closely related, others belonged to distinct molecular complexes. We conclude that the general assumption that one individual is predominantly colonized by one type of *S. aureus* does not apply to chronic wounds of EB patients. We consider this observation important, not only for EB patients, but also for other patients with chronic wounds in view of the potential risk for severe staphylococcal infections.

## Introduction

*Staphylococcus aureus* is a Gram-positive bacterium that colonizes ∼30% of the healthy human population [1]. However, under certain circumstances, *S. aureus* transforms from harmless commensal into a dangerous pathogen that can cause life-threatening invasive diseases [2]. Accordingly, *S. aureus* carriage has been associated with an increased risk of severe infections [3]. In most carriers, only one type of *S. aureus* is encountered, suggesting an intimate relationship between the carrier and the carried strain [3]. Nevertheless, the presence of multiple *S. aureus* types has been reported in healthy individuals and in patients with particular diseases, such as cystic fibrosis, atopic dermatitis and the genetic blistering disease epidermolysis bullosa (EB) [4]–[8]. The presence of multiple *S. aureus* types was found to be exceptionally high in EB patients with chronic wounds, who were shown to carry up to four *S. aureus* types at one time point of sampling [7]. Over longer periods of time, up to six alternating *S. aureus* types were isolated from these patients, and this high exposure to *S. aureus* seems to elicit strong antistaphylococcal immune responses [8]. Specifically, four major EB subtypes can be distinguished, namely EB simplex (EBS), junctional EB (JEB), dystrophic EB (DEB), and Kindler syndrome [9]. As shown in our previous studies, high-level wound colonization in EB patients by multiple types of *S. aureus* predominantly relates to the presence of chronic wounds, rather than the type of EB from which the investigated patients suffer [7], [8].

Notably, in most previous studies, the observed co-existence of multiple *S. aureus* types related to the analysis of swab samples from different body sites where one *S. aureus* colony was analyzed per swab sample [7], [8]. In only one study, multiple *S. aureus* colonies derived from individual nasal and perianal swap samples were analyzed, showing that children selected for elective surgery carried up to three *S. aureus* types at one particular body site [4]. To date, the co-existence of multiple *S. aureus* types has not been studied quantitatively and, due to the swab sampling methodology, nothing is known about the relative distances at which these co-existing *S. aureus* types are located from each other. Furthermore, little information is available on the genetic relatedness of co-existing *S. aureus* types and hence it is not known whether these are derived from in-patient evolution or the simultaneous acquisition from different sources [10]. The present studies were therefore aimed at investigating the co-existence of different *S. aureus* types in relation to their genetic relatedness and spatial distribution *in vivo*. This is only possible by investigating individuals that carry these *S. aureus* types on body sites that are readily accessible for direct sampling without disturbing the microbial topography. As shown here, such requirements are met by the chronic wounds of patients with EB [9], which are extensively colonized with *S. aureus* [7]. Briefly, we investigated the spatial distribution of *S. aureus* in the chronic wounds of five EB patients by replica plating of used bandages onto agar plates, and subsequent typing of multiple *S. aureus* colonies. As shown by fluorescence *in situ* hybridization (FISH), the typed colonies on the replica plates represent micro-colonies of *S. aureus* that already existed in the investigated wounds. Up to six co-existing *S. aureus* types were encountered in individual wounds, often in close proximity. While some adjacent *S. aureus* isolates were closely related, others belonged to distinct molecular complexes.

## Materials and Methods

### Ethical Approval

The authors declare that experiments using human materials were performed with institutional approval upon written informed patient consent, and with adherence to the Helsinki Guidelines. Specifically, the medical ethics committee (METc) of the University Medical Center Groningen (UMCG) approved of the collection of non-invasive samples from patients with EB on the basis of written informed patient consent. The required informed consent was obtained from all EB patients included in the present studies.

### Patients and Bacterial Isolates

In total, five patients with EB from the Dutch Epidermolysis Bullosa Registry (DEBR) were recruited in this study. Notably, the patient numbers 1, 14, 62, 63 and 64 used in this paper correspond to patient numbers in our previous publications [7], [8]. Patients 1 and 63 have JEB, and patients 14, 62 and 64 have DEB. Samples from three patients (62, 63, 64) were taken from three distinct wounds, the left and right anterior nares, and the throat of each patient using transswabs (MWE, Corsham, England). *S. aureus* was isolated from the obtained samples as previously described [7]. In addition, the bandages from one to four different wounds were collected from all patients. In case of patient 14, the bandages from two distinct wounds were collected three times at two-weekly intervals. All collected bandages were immediately replica-plated onto cysteine lactose electrolyte-deficient agar (CLED, oxoid) by gently pressing them for 5 to 10 s on the agar surface. To this end, bio assay plates were used (245×245×25 mm; Nunc). After 24 h of incubation at 37°C, 12 to 48 *S. aureus* colonies per wound/bandage were selected for further analysis. The isolation of *S. aureus* was confirmed by standard diagnostic methods.

### Molecular Characterization of*S. aureus*

All isolated *S. aureus* colonies were typed by Multiple-locus Variable Number of Tandem Repeats Fingerprinting (MLVF) as previously described [11]. Only identical patterns were regarded as the same subtype. Isolate clusters were delineated with a 60% similarity cutoff value. In addition, unique *S. aureus* MLVF types isolated from bandages of EB patients 62, 63 and 64 were characterized by Multiple Loci Variable Number of Tandem Repeats Analysis (MLVA) and *spa*-typing as previously described [7].

### Fluorescent*in situ* Hybridization

Bandages from EB patient 14 were used for FISH. Slides coated with gelatin-suspension (0.1% gelatin, 0.01% KCr(SO_4_)_2_.12H_2_O, Sigma) were pressed onto used bandages (15 s), air-dried, fixed with 96% ethanol (5 min), and stored at −20°C until further processing. FISH was performed as previously described with minor modifications [12,12]. Briefly, cells were fixed with 4% paraformaldehyde for 1 h at 4°C, and permeabilized with 2 U/ml lysostaphin. After dehydration with 96% ethanol, the cells were hybridized for 2 h at 50°C with the STAUR and EUB338 probes (5 ng/µl each) in hybridization buffer, and then washed for 10 minutes in wash buffer [12]. Fluorescence microscopy was performed with a Leica DM RXA microscope. The phase contrast and fluorescent images were merged using Adobe Photoshop CS4.

## Results and Discussion

### *S. aureus* Topography in Chronic Wounds

**Figure 1:**
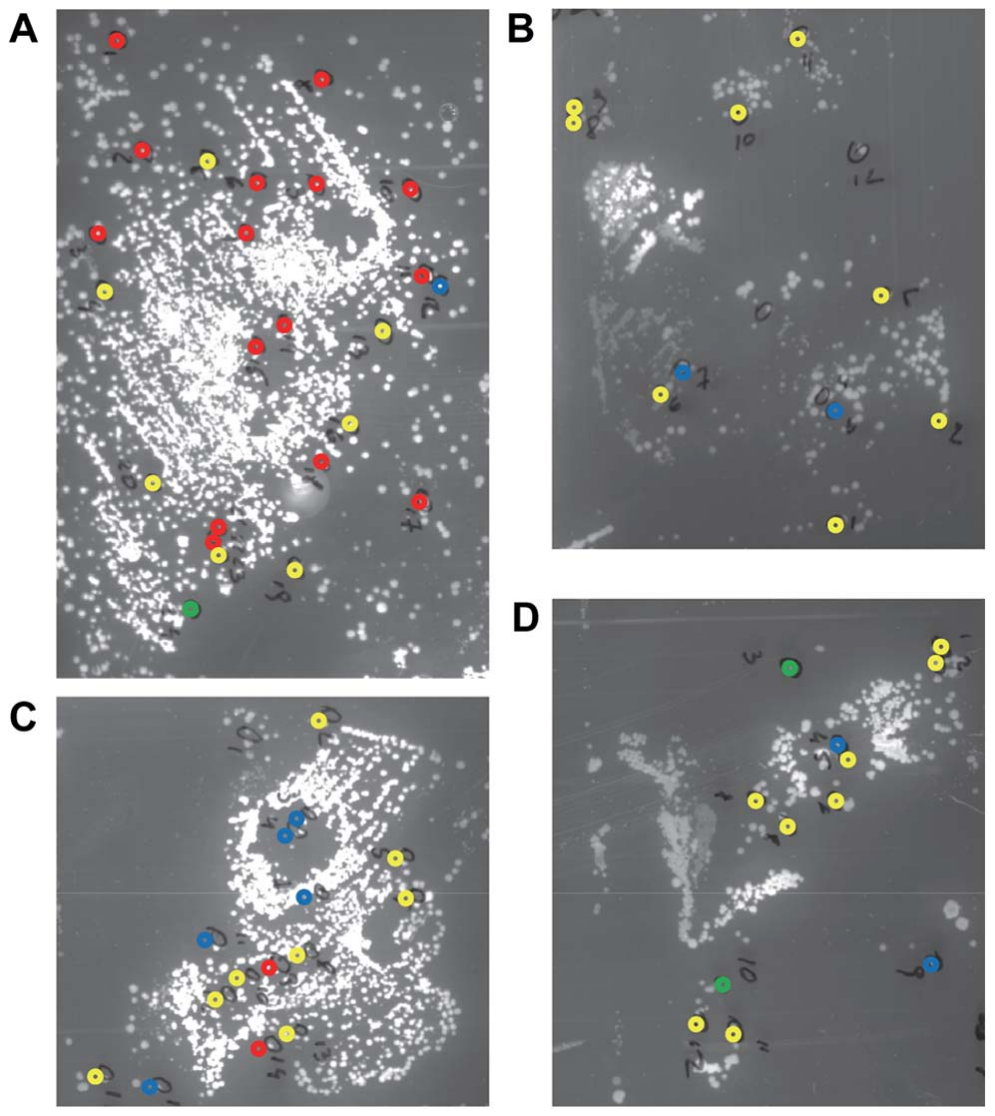
*S.* *aureus* chronic wound topography. Used bandages from four chronic wounds (A–D) of patient 64 were replica-plated onto CLED agar plates. The plates were incubated overnight at 37°C. Individual *S. aureus* colonies were isolated for typing by MLVF. *S. aureus* colonies with different MLVF types are indicated in different color codes: red, type 1; blue, type 2, yellow, type 3; green, type 4. Note that type 3 isolates belong to a very different molecular complex than type 1, 2 and 4 isolates (Table 1).

**Figure 2:**
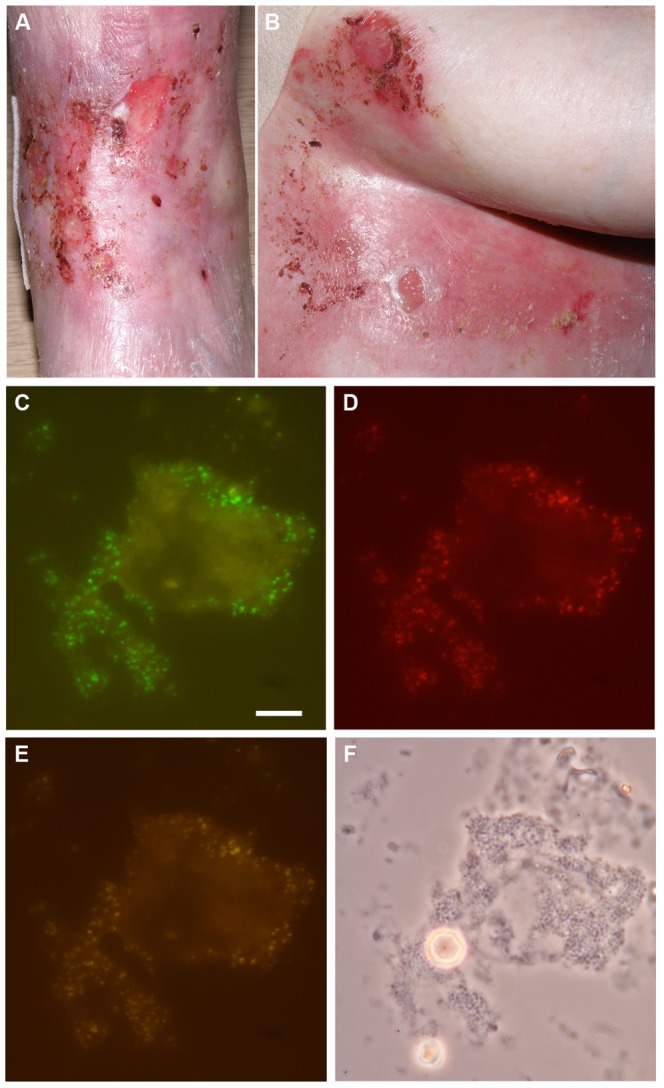
Chronic wounds contain microcolonies of*S.* *aureus*. To study the micro-topography of *S. aureus* in chronic wounds of patient 14 (A, B), a glass slide was pressed onto a freshly removed used bandage. The presence of *S. aureus* was visualized by FISH using an *S. aureus*-specific PNA probe. C, FISH with a universal bacterial PNA probe; D, FISH with an *S. aureus*-specific PNA probe; E, overlay of images C–D; F, phase contrast image. The white bar in panel C indicates 10 µm.

To determine the topography of *S. aureus* in chronic wounds of EB patients, used bandages of five patients were replica plated onto CLED agar. These bandages had covered the wounds for ∼24 h and they were replica plated immediately upon redressing of the wounds. The plates were then incubated overnight at 37°C. Depending on the patient and the investigated wounds, we observed either confluent growth or separate colonies at different densities. Where possible, regions of the plates with separate colonies (Figure 1) were directly used for further analysis by picking of colonies and subsequent species determination using standard diagnostic methods. In case of patient 62, only overlapping colonies were detectable. Therefore, these colonies were streaked on fresh plates so that individual colonies could be isolated for further analyses. The vast majority of the investigated colonies from all patients included in this study were shown to represent *S. aureus*. Next, we investigated whether such colonies were derived from individual cells or pre-existing micro-colonies in the investigated wounds. To this end, the bandages of one EB patient were pressed onto glass slides and FISH analyses were performed with an *S. aureus*-specific oligonucleotide probe (Figure 2). As a control, FISH was performed with a probe specific for bacteria in general. This confirmed that the vast majority of bacteria present in the investigated wounds were *S. aureus*. Importantly, these *S. aureus* cells were mostly present in micro-colonies, although individual *S. aureus* cells were also detectable. We therefore conclude that the bacterial wound topography as observed through replica plating of used bandages closely reflects the actual bacterial topography in the wounds that were dressed with the respective bandages. In those cases where confluent growth on plates was observed by replica plating, the bacteria in the wounds were apparently present in the form of biofilms [13].

### Co-existence of Distinct*S. aureus* Types in Chronic Wounds

**Figure 3:**
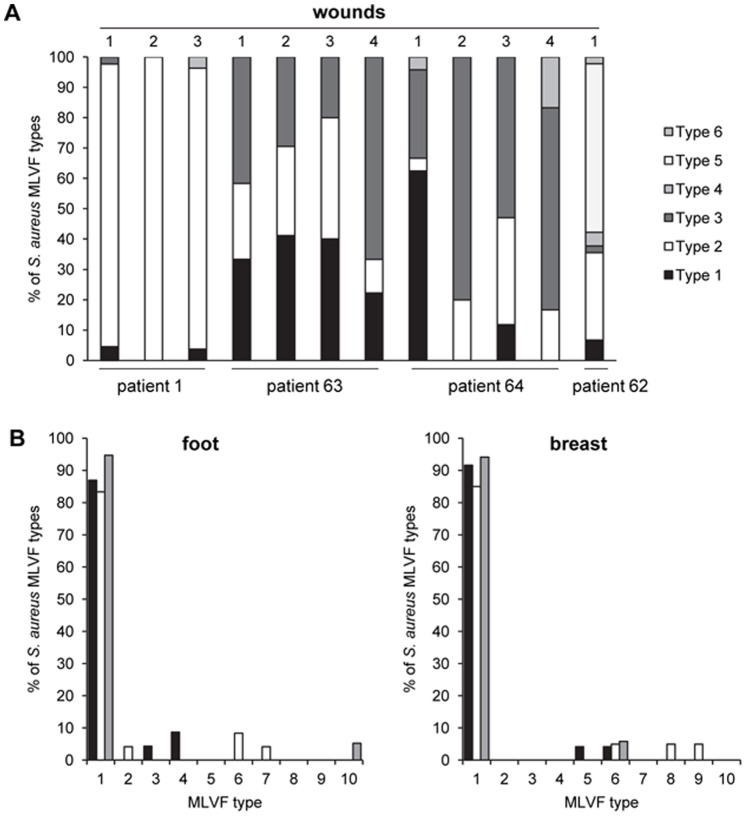
Relative numbers of MLVF types detected in the chronic wounds of five EB patients. A, The percentages of different *S. aureus* MLVF types detected in individual chronic wounds from patients 1, 62, 63 and 64 at one particular time point are indicated by bar diagrams. Different MLVF types are marked in black, white or grey shading. Note that, for matters of simplicity, the MLVF type numbers were arbitrarily attributed to different *S. aureus* types isolated from individual patients. Hence, distinct *S. aureus* types isolated from different patients can have the same type number. B, percentages of different *S. aureus* MLVF types detected in chronic foot and breast wounds from patient 14. Replica plating of used bandages was performed thrice at two-weekly intervals. Black bars mark the first, white bars the second and grey bars the third time point of sampling.

The presence of different co-existing *S. aureus* types in the investigated wounds was first assessed by MLVF. This technique allows the high-resolution typing of many *S. aureus* isolates in a very short period of time [11], which makes it ideal for the distinction of large numbers of isolates [14]–[16]. Per replica-plated bandage, we typed 12 to 48 colonies, and for each patient at least one replica-plated bandage was included from which 24 colonies were typed. In total, 368 *S. aureus* colonies were typed (36 to 85 colonies per patient at any particular time point of sampling), which yielded 27 distinct *S. aureus* MLVF types. Thus, we typed a substantially larger number of colonies per patient than the 20 colonies per patient that were previously recommended for studies with small numbers of carriers [17]. As shown in Figure 3A, we detected between two and six different *S. aureus* types in individual chronic wounds of four EB patients at one time point of sampling. By investigating the wound colonization in patient 14 over time, we detected even up to 10 different *S. aureus* types (Figure 3B). In the wounds of patients 1 and 14 dominant MLVF types were identified, which were accompanied by several less abundant MLVF types. On the other hand, less drastic differences were observed in the relative abundance of identified MLVF types in the wounds of patients 62, 63 and 64 (Figure 3A). Intriguingly, when the MLVF results were assessed in relation to the topography of typed *S. aureus* colonies, it became clear that different *S. aureus* types can exist in very close proximity (Figure 1). This conclusion was underscored by the observation that six different types of *S. aureus* were separated from overlapping colonies in the sampled wound of patient 62. To our knowledge this is the first time that the relative numbers of co-existing *S. aureus* types at one particular body site have been quantified, and that their topography has been mapped in detail. Also, the co-existence of different *S. aureus* types in close proximity has not been shown before.

**Figure 4:**
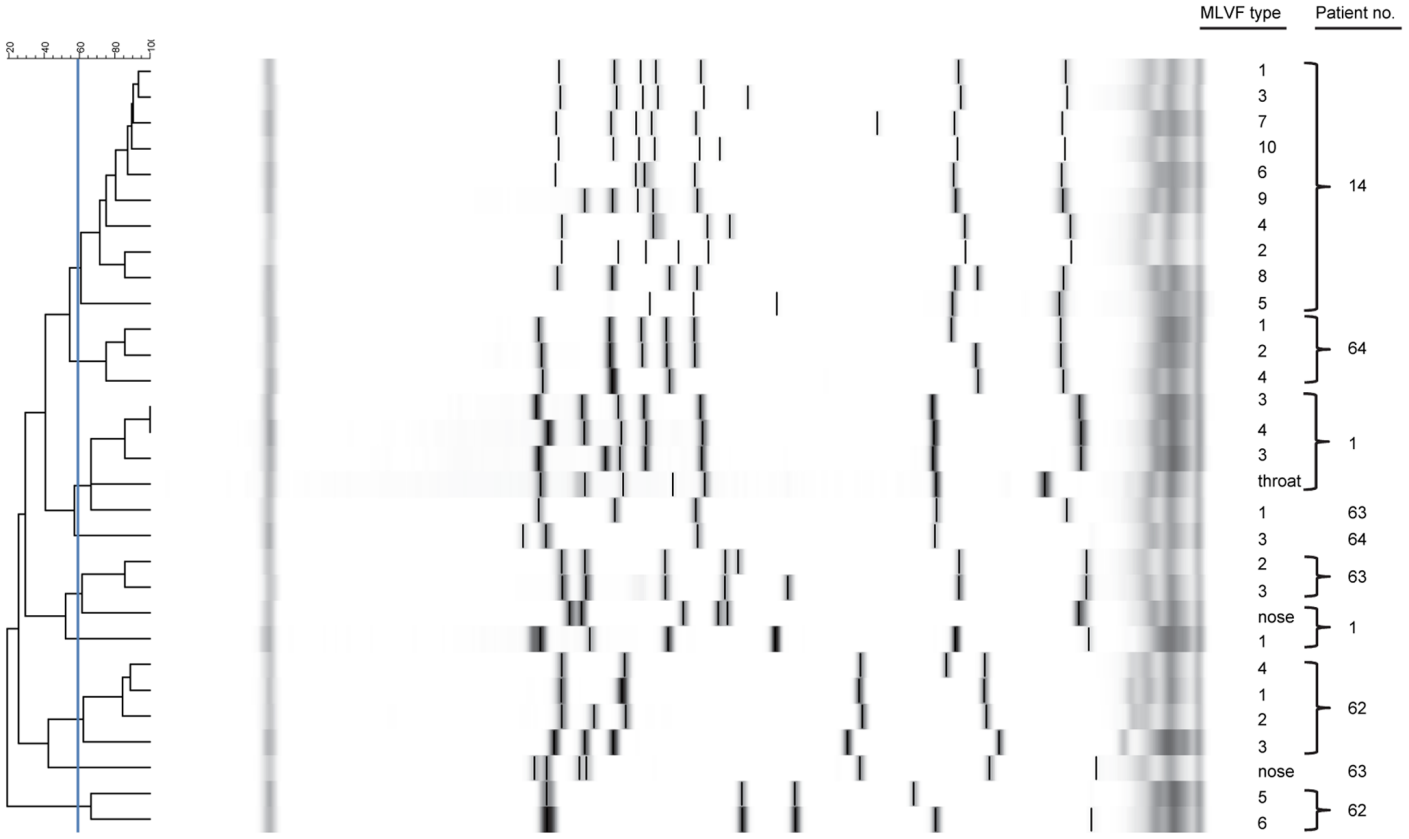
MLVF dendrogram of different*S.* *aureus* types isolated from chronic wounds of patients with EB. The dendrogram was generated using the Dice coefficient with Tolerance 0.6 and optimization 0.5. Clusters of isolates were generated with a cut-off value set to 60%. The MLVF types of individual *S. aureus* isolates are indicated in the dendrogram together with the number of the patient from which they were collected. Type numbers per patient correspond with the type numbers per patient in Figure 3.

To approximate the relatedness of the mapped *S. aureus* types, the results of our MLVF analyses were evaluated with GelCompar II (Applied Maths). As shown in the resulting dendrogram, the different *S. aureus* isolates from patient 14 all clustered together due to their similar MLVF patterns (Figure 4). This suggests that these isolates share a common ancestry and that, for example, the dominant MLVF type 1 has been most successful in colonizing the investigated wounds of patient 14. Smaller clusters of two to four related *S. aureus* MLVF types were also identified in the wounds of patients 1, 62, 63 and 64, which suggests that some of the staphylococci in wounds of these patients have recently evolved from a common ancestor. However, individual wounds of the latter patients also contained apparently unrelated MLVF types. Since this suggested that the latter isolates represent very different *S. aureus* types, they were further analyzed in detail by *spa*-typing and MLVA. These analyses indeed demonstrated that the wounds of patients 62, 63 and 64 contained *S. aureus* types that belong to distinct molecular complexes as defined by their *spa*- and MLVA-types (Table 1). Specifically, the MLVF types 1–4 versus types 5 and 6 of patient 62 belong to very different molecular complexes, and the same is true for types 1 versus 2 and 3 of patient 63, and types 1, 2, and 4 versus type 3 of patient 64. Some of the MLVA types encountered in wounds can actually also be recovered from the nares or throats of the respective patients (data not shown), but these body sites also contain other *S. aureus* types as shown in Figure 4 for patients 1 and 63.

**Table 1:** MLVA and spa typing of*S. aureus* isolates from chronic wounds of EB patients.

	MLVF	*spa*	MLVA		VNTR^a^ number							
			type	MC^b^	09_01	61_01	61_02	67_01	21_01	24_01	63_01	81_01
Patient 62	Type 1	t254^I^	1298	15	7	5	2	1	1	9	99	4
	Type 2	t254^I^	1298	15	7	5	2	1	1	9	99	4
	Type 3	t6993^I^	3393	none	7	5	2	1	1	8	99	4
	Type 4	t254^I^	1298	15	7	5	2	1	1	9	99	4
	Type 5	t7473^II^	532	45	11	3	3	4	1	12	1	5
	Type 6	t9968^II^	45	45	11	3	3	4	1	11	1	5
Patient 63	Type 1	t002^I^	23	5	14	2	1	3	2	11	7	5
	Type 2	t078^II^	2049	none	12	0	2	2	1	10	6	3
	Type 3	t078^II^	2049	none	12	0	2	2	1	10	6	3
Patient 64	Type 1	t9969^I^	3391	none	14	1	1	1	1	10	7	5
	Type 2	t509^I^	3392	none	14	1	1	1	1	9	6	5
	Type 3	t015^II^	45	45	11	3	3	4	1	11	1	5
	Type 4	t509^I^	3392	none	14	1	1	1	1	9	6	5

a VNTR, Variable Number of Tandem Repeats.

b MC, molecular complex.

Per patient, the isolates with closely related *spa*-types have been marked by ^I^ or ^II^.

The typing of *S. aureus* isolates by MLVF and MLVA is based on variable number of tandem repeat (VNTR) loci. Specifically, the number of tandem repeat sequences at different genomic loci are determined by multiplex PCR and subsequently the numbers of repeat units in the resulting amplicons are either approximated by (microfluidic) electrophoresis (MLVF) or exactly defined by capillary electrophoresis on an automatic DNA sequencer (MLVA) [15]. The advantage of MLVF is that it is simple, cheap, fast and easy in use. However, the resulting data cannot be compared directly between different laboratories, as the MLVF amplicons are monitored as banding patterns. MLVA is more costly and time-consuming than MLVF, but it does define the precise numbers of repeats within each investigated VNTR, thereby generating high-resolution portable data that can be directly compared to reference databases. As shown in our present and previous analyses [14]–[16], the outcomes of MLVF and MLVA are compatible, although MLVF had a clearly higher resolution in the present studies than MLVA (Table 1). This was an important advantage of MLVF as it allowed us to pinpoint evolving *S. aureus* types in the wounds of EB patients. Contrary to MLVF and MLVA, *spa*-typing is a single-locus typing approach. Its important advantages are that it is highly reproducible, highly standardized and fully portable due to implementation of the Ridom database (http://spaserver.ridom.de). Accordingly, *spa*-typing is currently the most useful tool for typing *S. aureus* isolates at the national and international levels [10], [18]–[22]. Importantly, in the present study, also the outcomes of MLVA and *spa*-typing were fully compatible (Table 1), and this underpins the conclusion that the chronic wounds of patients 62, 63 and 64 harbor distinct types of *S. aureus*. It should be noted that the clinical relevance of the subtypes identified by MLVF is presently not clear. However, these subtypes seem to represent evolving isolates some of which may eventually happen to have a higher fitness than their parental type. This view would be in line with the fact that different isolates of particular clonal clusters of *S. aureus* according to *spa*- or MLVA type may have very different molecular characteristics, epidemiologies of colonization and infection, and antimicrobial resistance profiles.

In conclusion, our present study shows that chronic wounds of individual EB patients can be colonized by several distinct *S. aureus* types. As reported here for the first time, these distinct *S. aureus* types can form micro-colonies that are located in close proximity (Figure 1). This implies that these different types are not mutually exclusive. It thus seems that the dogma that one individual is usually colonized by one *S. aureus* type does not apply to the wounds of patients with EB. We consider this finding important, not only for patients with EB, but also for other patients with chronic wounds where a similar colonization by more than one *S. aureus* type might occur, because *S. aureus* is known to interfere with wound healing and poses a significant risk factor for infections and invasive disease [23].
